# Association study of the polymorphisms rs2228611 of the *DNMT1* gene and rs1569686 of the *DNMT3B* gene with bladder cancer development in a sample of the Algerian population

**DOI:** 10.22099/MBRC.2023.48569.1881

**Published:** 2024

**Authors:** Zohra Touala-Chaila, Rym-Khadidja Abderrahmane, Slimane Kerroumi, Mostefa-Jamel Yousfi, Djebaria-Naima Meroufel, Abdallah Boudjema

**Affiliations:** 1 Université Des Sciences Et de La Technologie d’Oran Mohamed-Boudiaf USTOMB El Mnaouar, BP 1505, Bir El Djir, 31000 Oran, Algerie. Laboratoire Génétique Moléculaire Et Cellulaire (LGMC) USTOMB; 2 Etablissement Hospitalier Universitaire d'Oran EHU 1 Novembre 1954, service Chirurgie Urologique, Oran, Algerie

**Keywords:** Bladder cancer (BC), rs2228611, rs1569686, DNMT1, DNMT3B

## Abstract

Bladder cancer (BC) is a multifactorial disease with a poorly understood main cause. In this study, we aimed to evaluate the effect of the polymorphisms rs2228611 of the DNMT1 gene and rs1569686 of the DNMT3B gene on the susceptibility to develop Bladder Cancer in the Algerian population. A case-control study design was adopted, with DNA samples of 114 BC patients and 123 healthy controls. We found that the rs2228611 of the DNMT1 gene was strongly associated with an increased risk of BC development under genetic models: Codominant AG *vs*. GG (OR=2.54, 95% CI=1.21-5.51, adj p=0.015) and dominant AA+AG *vs*. GG (OR=2.24, 95% CI=1.12-4.60, adj p=0.023). However, no statistically significant association was observed between the rs1569686 of the DNMT3B gene and the predisposition to BC. To the best of our knowledge, this is the first peer-reviewed study to evaluate the effect of the rs2228611 polymorphism on bladder cancer occurrence. Our results suggest that the rs2228611 might be a potential biomarker for BC development risk. Additional studies are needed to validate our findings.

## INTRODUCTION

According to International Agency for Research on Cancer, a total of 57,3278 new cases of bladder cancer (BC) were diagnosed worldwide, with 21,2536 deaths in 2020. These diagnostic numbers may increase to 991,000 cases by the year 2040 http://gco.iarc.fr/today/home https://tinyurl.com/mpnu4zyp. Over three-quarters of new cases of bladder cancer are observed in men. Indeed, bladder cancer is the sixth most common cancer in men and 17th in women worldwide [1]. In Algeria, bladder cancer is the fifth most common cancer, with 3,201 new cases and 1,861 deaths https://gco.iarc.fr/today/fact-sheets-populations .

Smoking is one of the main risk factors for BC, contributing to 30-40% of all cases of urothelial carcinoma. The second major risk factor for BC is exposure to carcinogenic substances such as aromatic amines, which have been linked to 5-10% of all cases of BC. Other factors include gender, with a higher incidence rate of BC in men than in women, and advanced age, with a median diagnosis of 73 years [2]. In addition to environmental factors, genetic predisposition to BC was also importat. In a family in which a man and his three sons developed carcinoma of the bladder. The family environment contained no chemical elements suspected of causing bladder carcinogenesis, leading to the possibility that the disease was due to a genetic mechanism [3].

Several genes have been reported to be involved in the development of bladder cancer, including *FGFR3*, RB1, *HRAS*, *TP53* and *TSC1*.These genes play an important role in cell division regulation, through their ability to control the rate of this division [4]. Besides classical genetic abnormalities, an epigenetic component in bladder cancer pathology has been demonstrated. Indeed, epigenetics is generally considered to be "the study of changes in gene function that are inherited mitotically and/or meiotically and that do not involve changes in DNA sequence" [5].

Many studies have reported the potential role of DNA methylation markers as urinary biomarkers for the diagnosis of bladder cancer. DNA hypermethylation is considered to be one of the earliest events in urothelial carcinogenesis. Indeed, hypermethylation of the promoter regions of tumor suppressor genes can lead to inactivation of their function, thus contributing to the development of cancer. DNA methylation analysis is therefore a promising tool for the early detection of bladder tumors [6].

A family of DNA methyltransferase enzymes (DNMT1, DNMT3a, DNMT3b and DNMT3L) are responsible for the establishment and faithful maintenance of DNA methylation profiles in mammalian cells. The DNMT3a and DNMT3b are so-called de novo DNMT enzymes, which establish the methylation patterns during embryonic development, genomic imprinting and X chromosome inactivation [7]. Whereas the *DNMT1* gene codes for an enzyme responsible for maintaining methylation patterns after DNA replication, and shows a preferential response to hemi-methylated DNA https://www.ncbi.nlm.nih.gov/gene/1786. In the present study, we aim to evaluate possible implication of SNPs of the DNA methylation genes *DNMT1* and *DNMT3B* in the occurrence of bladder cancer in the Algerian population. For this purpose, 2 polymorphisms were chosen: rs2228611 of the *DNMT1* gene, and rs1569686 of the *DNMT3B *gene.

## MATERIAL AND METHODS

**Study population: **A total of 114 patients were recruited at Etablissement Hospitalo-Universitaire d'Oran (EHU-Oran), Service Urologie. A total of 123 cancer-free normal control subjects were recruited from various departments including: department d’Orthopédie et de Traumatologie, le Centre de Transfusion Sanguine EHU d’Oran and Oran home for the elderly. All the subjects were of Algerian origin. Overall, the study was carried out in accordance with Declaration of Helsinki. Participants gave their informed consent to the researcher and agreed to participate in the study. 

**DNA extraction: **A peripheral blood sample was taken from participants and stored at -20°C until DNA extraction. The samples were extracted for genomic DNA using the Blood DNA Extraction Kit (WiraGen) according to the manufacturer's instructions, and the NaCl "Salting out" technique adapted to the protocol of Miller et al. [8].

**Genotyping of rs2228611/1569686: **The rs2228611 of the *DNMT1* gene, located on exon 17, and the rs1569686 of the *DNMT3B* gene, located at the promoter, were amplified by Polymerase Chain Reaction (PCR). We used the following primers: F:5’- TATGTTGTCCAGGCTCGTCTC 3’//R: 5’-GTACTGTAAGCACGGTCACCTG3’/ and F:5’-GAGGTCTCATTATGCCTAGG3’//R:5’-GGGAGCTCACCTTCTAGAAA-3’ for rs2228611 and rs1569686 respectively. Overall, PCR was carried out in a final volume of 20 µl according to the manufacturer's recommendation (FIREPOL Master Mix Ready to load 5X PCR reaction mix du fabricant SOLIS BIODYNE). The expected amplimer size of the SNP rs2228611 of the *DNMT1* gene is 260bp.

The PCR program used included an initial denaturation step at 95°C for 5 minutes, followed by 35 cycles of denaturation at 95°C for 30 seconds, hybridization at 63°C (depending on loci) for 30 seconds, elongation at 72°C for 40 seconds, and a final elongation step at 72°C for 10 minutes [9]. In order to have a 225 bp amplimer, the PCR of the rs1569686 of the *DNMT3B* gene was carried out over 35 cycles of 30 seconds at 95°C, 45 seconds at 56°C and 45 seconds at 72°C, with an initial denaturation step of 5 min at 95°C and a final elongation step of 10 min at 72°C [10]. RFLP (Restriction Fragment Length Polymorphism) was used to detect polymorphisms in the *DNMT1* and *DNMT3B* genes, using the enzymes BsmAI and PvuII respectively.Digestion was carried out according to the manufacturer's instructions (NEBcloner, BioLAbs). To make a final volume of 25µl, we took 2.5ul of Buffer 10x, 0.5 µl of BsmAI enzyme, 10µl of PCR product, and 12µl of water. The mixture was incubated at 55°C for 15 minutes. Similar volumes were taken for *Pvu*II, except that the incubation temperature was 37°C for 15 minutes. The expected digestion product sizes for the *DNMT1* gene SNP are: 232pb- 28pb for the wild homozygote and 108pb-124pb-28 for the mutated homozygote. Whereas the expected digest product sizes of the SNP of the D*NMT3B* gene are: 225bp for the wild-type allele, and 132bp- 93bp for the mutated allele. Both amplification and digestion products were visualized by 1% agarose gel electrophoresis.

**Statistical analysis: **Hardy-Weinberg Equilibrium (HWE) estimation was determined in controls by the Exact Test for HWE. The OR and 95% CI were calculated in order to assess the association between the SNPs rs2228611, rs1569686 of the *DNMT1* and *DNMT3B* genes respectively with bladder cancer occurrence.

Given the fact that the Bonferroni correction is used to control false positives (Type I error), it can become very conservative as the number of tests increases. This, in turn, increases the risk of generating false negatives (type II error). We therefore chose to set the statistical significance level of the association at<0.05. Statistical tests were performed using R software (Windows) 4.2.3/ RStudio: 2023.03.0+386. Packages («genetics" for HWE test, "glm2" for univariate and multivariate binary logistic regression, “dplyr” to obtain the proportions). The "epiR" and "epitools" packages were used to confirm the results of univariate binary logistic regression. We used the "mean" and "SD" functions respectively to calculate the mean and standard deviation.

The association was tested using multivariate binary logistic regression, taking into account the following confounding factors: sex, age and smoking. The association was tested according to genetic models: codominant, dominant, recessive and overdominant. A p<0.05 was considered significant.

## RESULTS

In this study, the distribution of age, sex and tobacco consumption was compared between the case group and the control group as shown in Table 1. The majority of cases and controls were men, with an average age of 62 for cases and 60 for controls. In the present study, most participants answered "no" to the smoking question, representing a percentage of 72% in cases and 64% in controls. 

There was no deviation from Hardy Weinberg equilibrium for the two polymorphisms (exact test p>0.05) (Table 2). As shown in Table 2, the A and T alleles of rs2228611 and rs1569686 respectively are considered minor alleles due to their frequency in controls. 

**Table 1:** Description of study participants (cases/controls)

**Variables**					**Cases=114 n (%)**			**Control=123 n (%)**	
**Sex**		**Men**			108 (95%)			97 (79%)	
			**Women**			6 (5%)		26 (21%)		
**Tabac**		**Yes**			31 (27.1%)			41 (33.3%)	
			**No**			82 (72%)		79 (64.2%)		
		**NA** ^a^			1 (0.9%)			3 (2.4%)	
**Age (years)**		**Mean (SD)** ^b^			62.48 (14.31)			60.21 (11.95)	

**a**: Not Available; **b**: Standard Deviation

**Table 2:** Genotype distributions of the rs2228611 and rs1569686 in cases and controls

**SNPs**		**Allele Frequency**		**Genotype**			**Exact Test for Hardy-Weinberg Equilibrium**
***DNMT1*** **rs2228611**	**Case=101/114**	**A**	**G**	**AA**	**AG**	**GG**	--
		0.55	0.44	28	56	17	
	**Control=106/123**	0.47	0.56	26	48	32	p = 0.3364
***DNMT3B*** **rs1569686**	**Case=99/114**	**T**	**G**	**TT**	**TG**	**GG**	--
		0.48	0.51	24	49	26	
	**Control=110/123**	0.44	0.55	22	54	34	p = 1

*: p-value_(HWE)_>0.05  population in equilibrium.

A statistically significant association was found between the rs2228611 of the *DNMT1* gene and the occurrence of bladder cancer (Table 3) under the genetic models: codominant AG *vs.* GG (OR=2.54; 95% CI= 1.21-5.51, adj p=0.015,) and dominant AA+AG *vs.* GG (OR=2.24; 95% CI=1.12-4.60, adj p=0.023). However, we found no statistically significant association between the SNP rs1569686 of the *DNMT3B* gene and bladder cancer development in our population (Table 3).

## DISCUSSION

Studies investigating the association between polymorphism rs2228611 of the *DNMT1* gene and rs1569686 of the *DNMT3B* gene and bladder cancer are rare almost none. In the present study, the polymorphism rs1569686 have no statistically significant association with the development of BC. These results are in concordance with the only reviewed study carried out in the Taiwanese population [11]. and confirm our earlier findings from a meta-analysis [12]. However, the frequency of the T allele found in controls is 0.4, compared with 0.9 in the study of Chung et al. This results can be explained by the ethnical differences between the two populations, or by the limited number of participants. For this reason, additional studies with larger sample sizes are needed to confirm or refute the results of the present study in both the Algerian and North African populations. 

In another Taiwanese study on nasopharyngeal carcinoma, no association was found between rs1569686 and the occurrence of the disease, suggesting that *DNMT3B* may not play a role in the hypermethylation of many tumor suppressor genes during carcinogenesis in Taiwanese [13]. Furthermore, the results of various meta-analyses have demonstrated that the polymorphism rs1569686 of the *DNMT3B* gene could play a protective role against lung cancer and colorectal cancer in Asians and in the Azerbaijani population [14, 15] and against gastric carcinogenesis [16]. 

**Table 3:** Statistical analysis of the association between polymorphisms rs2228611 of the *DNMT1* gene and rs1569686 of the *DNMT3B* gene with bladder cancer susceptibility

**Gene SNP**	**Genetic model**	**Results**			
		**Univariate binary logistic regression (crude OR)**	**Crude ** ***p-value***	**Multivariate binary logistic regression (Adjusted OR for age, sex, smoking)**	**Adjusted** ***p-value***
*DNMT1* rs2228611	codominant model AA vs.GG	OR=2.02 95% CI (0.92-4.54)	0.081	OR=1.79, 95% CI (0.75-4.32)	0.188
	**codominant model ** **AG vs.GG**	**OR=2.19 ** **95% CI (1.09-4.50)**	**0.028**	**OR=2.54** **95% CI (1.21-5.51)**	**0.015**
	**dominant model** **AA+AG vs. GG**	**OR=2.13** **95% CI (1.11-4.23)**	**0.025**	**OR=2.24** **95% CI (1.12-4.60)**	**0.023**
*DNMT3B* rs1569686	codominant model TT vs.GG	OR=1.42 95% CI (0.66-3.10)	0.367	OR=1.59 95% CI (0.66-3.89)	0.301
	codominant model TG vs.GG	OR=1.18 95% CI (0.62-2.26)	0.601	OR=1.14 95% CI (0.58-2.22)	0.698

According to our research, the present study is the first reviewed study to assess the association between the rs2228611 polymorphism of the *DNMT1* gene and bladder cancer risk in the Algerian population and worldwide. Chi-Jung Chung et al., worked on the same SNP in a poster presentation [17]. Our study results showed a significant association between the rs2228611 of the *DNMT1* gene and the occurrence of bladder cancer in the study population under the genetic models: Codominant model AG *vs. *GG and Dominant model AA+AG *vs.* GG. 

These findings are in agreement with the results of the Hao Li et al. meta-analysis, which demonstrated that the rs2228611 (G/A) is a risk factor and is significantly associated with breast cancer risk [18]. In addition, it has been shown that rs2228611 is associated with an increased risk of developing ovarian cancer in the Polish population [19]. However, our results are discordant with the meta-analysis of Honggjia et al. where there was no statistically significant association between this SNP and the occurrence of gastric cancer [16]. 

The process of DNA methylation is essential for gene expression. The writing and patterning of DNA methylation in mammals is carried out by DNA methyltransferases (DNMTs), in particular *DNMT1*, *DNMT3A* and *DNMT3B*. Recent data show that DNMTs abnormalities are involved in tumor transformation and progression, indicating that epigenetic perturbations caused by DNMTs abnormalities are associated with tumorigenesis [20].

When DNA methylation patterns are established early in embryogenesis, they are maintained in somatic cells in a cell-type-specific manner. During each cycle of DNA replication, DNA becomes hemimethylated, as only the CpGs of the parental strand remain methylated, while the CpGs of the newly replicated daughter strand are not methylated. To restore CpG methylation symmetry and retain specificity, the activity of the maintenance DNA methyltransferase recognizes hemimethylated CpGs and methylates the corresponding CpGs on the daughter strand. Biochemical, cellular and genetic evidence suggests that *DNMT1* is the principal maintenance methyltransferase [21]. The rs2228611 of *DNMT1* is a G-A transition, translating into a synonymous variation (CCG→CCA, Proline→Proline). 

 The SNPinfo bioinformatics tool predicts that rs2228611 is located in the exonic splicing enhancer (ESE) region [22], which was confirmed by Saradalekshmi et al. Indeed, this SNP could have a splicing regulatory function, since G/A results in the loss of three exonic splicing enhancer-binding motifs [23]**.** Meanwhile, Cheng et al. used ESEfinder analysis to determine the effect of rs2228611 on altered spermatogenesis, and found that the rs2228611 is located in the binding motifs of splicing factors SRSF1, SRSF2, SRSF5 and SRSF6. If the A allele is present, the binding motifs of SRSF1 and SRSF6 disappear, which may reduce the hnRNA (pre-mRNA) splicing activity. Consequently, they believe that the AA genotype (homozygosity of the A allele) may lead to reduced *DNMT1* expression, which may lead to altered spermatogenesis [24]. Based on the bioinformatics studies cited above, we suggest that the rs2228611 may lead to dysfunction of the DNMT1 enzyme, which can lead to hypomethylation of bladder cancer cell DNA because of its deficient activity.

Furthermore, there are several elements indicating that the codon optimality is a major determinant of mRNA stability in yeast. Bioinformatics analysis shows a strong correlation between the percentage of optimal codons and mRNA half-life. For instance, mRNAs with less than 40% optimal codons have a median half-life of 5.3 minutes, while mRNAs with more than 70% optimal codons have a median half-life of 20.1 minutes. For example, the Alanine codon GCU was highly enriched in stable transcripts while its synonymous codons, GCG and GCA were preferentially present in unstable transcripts. They observed remarkable differences in ribosome clearance when mRNAs encoding the same polypeptide were composed of optimal or non-optimal codons. These differences may reflect the additive effects of many small ribosomal hesitations at non-optimal codons [25]. 

In the present study, we may assume that the substitution of G to A generates the CCA codon, seen as a non-optimal codon, which gives rise to a less stable mRNA with a shorter lifespan than the optimal CCG codon. On the other hand, tumor cells are characterized by rapid, anarchic proliferation, which greatly increases the need for dNTPs. These latter are generated by the One-Carbon Metabolism pathway [26]. Indeed, Cao et al. observed that there is a kinetic regulatory mechanism controlling the reaction rates of one-carbon metabolism processes, which become competitive when their shared resources are limited, particularly when nucleotide synthesis rates are raised.The combination of this regulatory mechanism with rapid nucleotide synthesis (folate cycle) and reduced synthesis of CH3 methyl groups (methionine cycle) explains why there is almost universal evidence of overall reduced DNA methylation in cancer [27]. Based on these data, we suggest the following hypothesis to explain the global hypo methylation of the genome during the bladder tumorigenesis process induced by rs2228611.

Cell cycle homeostasis disruption by oncogene and tumor suppressor gene bias will cause uncontrollable replication of cell division. The latter is characterized by DNA replication that requires unstoppable synthesis of dNTPs through the One carbone metabolism pathway (folate cycle), to the detriment of the methionine cycle responsible for generating the CH3 methyl group. DNA methylation is also influenced by the low activity of the DNMT1 enzyme in the presence of the rs2228611 polymorphism, which acts on DNMT1 mRNA alternative splicing factor binding motifs. Finally, mRNA lifespan has been shown to be strongly correlated with optimal codon levels. In the case of rs2228611, mRNA lifespan is relatively short due to the presence of the non-optimal codon caused by the G<A substitution, resulting in a decrease in *DNMT1* expression responsible for the maintenance of DNA methylation.

This study must be considered in the context of certain limitations: The sample size needs to be increased to ensure representativeness and statistical power. Due to the lack of detailed data on tobacco consumption in the control group, we had to accept a yes/no response from the case group, which made it difficult to carry out a detailed analysis on the frequency of tobacco consumption, which is an important factor in the onset of the disease.

### Acknowledgement:

We would like to thank Dr. MESSAL-DJELTI Ahlem Nora, Dr. ABID Ghania, Pr. ABERKANE Meriem and Miss AMMOUR Amina, PhD student, for sharing their control DNA samples with us.

### Conflict of Interest:

The authors report no conflict of interest.

### Authors’ contribution:

ZTC: contributed to conceptualization, methodology, contributed to  writing the original draft preparation, data collection, data analysis and interpretation. RKA: contributed to conceptualization, methodology. was involved in data collection and manucript preparation and correction. SK: was involved in study conception, data and samples collections. MJY: contributed to facilitating samples collection for the study and supervised the project. DNM: was involved in study conception, data collection. Contributed to supervision. AB: contributed to supervision.

## References

[1] Jubber I, Ong S, Bukavina L, Black PC, Compérat E, Kamat AM, Kiemeney L, Lawrentschuk N, Lerner SP, Meeks JJ, Moch H, Necchi A, Panebianco V, Sridhar SS, Znaor A, Catto JWF, Cumberbatch MG (2023). Epidemiology of bladder cancer in 2023: A systematic review of risk factors. Eur Urol.

[2] Halaseh SA, Halaseh S, Alali Y, Ashour ME, Alharayzah MJ (2022). A Review of the Etiology and Epidemiology of Bladder Cancer: All You Need To Know. Cureus.

[3] Fraumeni Jr JF, Thomas LB (1967). Malignant bladder tumors in a man and his three sons. JAMA.

[4] Zhang X, Zhang Y (2015). Bladder cancer and genetic mutations. Cell Biochem Biophys.

[5] Dupont C, Armant DR, Brenner CA (2009). Epigenetics: definition, mechanisms and clinical perspective. Semin Reprod Med.

[6] Hentschel AE, Beijert IJ, Bosschieter J, Kauer PC, Vis AN, Lissenberg-Witte BI, van Moorselaar RJA, Steenbergen RDM, Nieuwenhuijzen JA (2022). Bladder cancer detection in urine using DNA methylation markers: a technical and prospective preclinical validation. Clin Epigenetics.

[7] Murphy TM, Mullins N, Ryan M, Foster T, Kelly C, McClelland R, O’Grady J, Corcoran E, Brady J, Reilly M, Jeffers A, Brown K, Maher A, Bannan N, Casement A, Lynch D, Bolger S, Buckley A, Quinlivan L, Daly L, Kelleher C, Malone KM (2013). Genetic variation in DNMT3B and increased global DNA methylation is associated with suicide attempts in psychiatric patients. Genes Brain Behav.

[8] Miller SA, Dykes DD, Polesky HF (1988). A simple salting out procedure for extracting DNA from human nucleated cells. Nucleic Acids Res.

[9] Khatami F, Noorinayer B, Ghiasi S, Mohebi R, Hashemi M, Zali MR (2009). Lack of effects of single nucleotide polymorphisms of the DNA methyltransferase 1 gene on gastric cancer in Iranian patients: a case control study. Asian Pac J Cancer Prev.

[10] Coppedè F, Ricciardi R, Denaro M, Rosa AD, Provenzano C, Bartoccioni E, Baggiani A, Lucchi M, Mussi A, Migliore L (2013). Association of the DNMT3B -579G>T polymorphism with risk of thymomas in patients with myasthenia gravis. PLos One.

[11] Chung CJ, Chang CH, Liu CS, Huang CP, Chang YH, Chien SN, Tsai PH, Hsieh HA (2014). Association of DNA methyltransferases 3A and 3B polymorphisms, and plasma folate levels with the risk of urothelial carcinoma. PLoS One.

[12] Touala-Chaila Z, Abderrahmane RK, Benseddik K, Meroufel DN (2022). A meta-analysis on the susceptibility to the development of bladder cancer in the presence of DNMT3A, DNMT3B, and MTHFR gene polymorphisms. Afr J Urol.

[13] Chang KP, Hao SP, Tsang NM, Chang YL, Cheng MH, Liu CT, Lee YS, Tsai CL, Lee TJ, Wang TH, Tsai CN (2008). Gene expression and promoter polymorphisms of DNA methyltransferase 3B in nasopharyngeal carcinomas in Taiwanese people: a case-control study. Oncol Rep.

[14] Zhang Y, Xu H, Shen Y, Gong Z, Xiao T (2015). Association of DNMT3B -283 T>C and -579 G>T polymorphisms with decreased cancer risk: evidence from a meta-analysis. Int J Clin Exp Med.

[15] Bayramov B, Bayramov N, Aslanov H, Abbasov M, Mammadova S, Eynullazada K, Gahramanova F, Yagublu V (2022). Investigation of the DNMT3B–579 G>T promoter polymorphism in patients with colorectal cancer in an Azerbaijani population. Asian Pac J Cancer Prev.

[16] Li H, Li W, Liu S, Zong S, Wang W, Ren J, Li Q, Hou F, Shi Q (2016). DNMT1, DNMT3A and DNMT3B polymorphisms associated with gastric cancer risk: A systematic review and meta-analysis. EBioMedicine.

[17] Chung CH, Chang CH, Liu CS, Chang YH, Chung MC The effect of cigarette smoking and DNA methyltransferase genes polymorphisms on urothelial carcinoma risk in Taiwan.

[18] Li H, Liu Jw, Sun LP, Yuan Y (2017). A meta-analysis of the association between DNMT1 polymorphisms and cancer risk. Biomed Res Int.

[19] Mostowska A, Sajdak S, Pawlik P, Lianeri M, Jagodzinski PP (2013). DNMT1, DNMT3A and DNMT3B gene variants in relation to ovarian cancer risk in the Polish population. Mol Biol Rep.

[20] Zhang W, Xu J (2017). DNA methyltransferases and their roles in tumorigenesis. Biomarker Res.

[21] Veland N, Chen T, Tollefsbol TO (2017). Chapter 2-Mechanisms of DNA Methylation and Demethylation During Mammalian Development. Handbook of Epigenetics (Second Edition).

[22] Jia Z, Wu X, Cao D, Wang C, You L, Jin M, Wen S, Cao X, Jiang J (2016). Polymorphisms of the DNA methyltransferase 1 gene predict survival of gastric cancer patients receiving tumorectomy. Dis Markers.

[23] Saradalekshmi KR, Neetha NV, Sathyan S, Nair IV, Nair CM, Banerjee M (2014). DNA methyl transferase (DNMT) gene polymorphisms could be a primary event in epigenetic susceptibility to schizophrenia. PLoS One.

[24] Cheng P, Chen H, Zhang RP, Liu S, Zhou-Cun A (2014). Polymorphism in DNMT1 may modify the susceptibility to oligospermia. Reprod Biomed Online.

[25] Presnyak V, Alhusaini N, Chen YH, Martin S, Morris N, Kline N, Olson S, Weinberg D, Baker KE, Graveley BR, Coller J (2015). Codon optimality is a major determinant of mRNA stability. Cell.

[26] Corbin JM, Ruiz-Echevarría MJ (2016). One-carbon metabolism in prostate cancer: The role of androgen signaling. Int J Mol Sci.

[27] Cao S, Zhu X, Zhang C, Qian H, Schuttler HB, Gong J, Xu Y (2017). Competition between DNA methylation, nucleotide synthesis and anti-oxidation in cancer versus normal tissues. Cancer Res.

